# Urokinase-type plasminogen activator blockade ameliorates experimental colitis in mice

**DOI:** 10.1038/s41598-023-29824-1

**Published:** 2023-02-18

**Authors:** Yoshifumi Kida, Toshiya Okahisa, Yasushi Sato, Masahiro Bando, Shota Fujimoto, Beibei Ma, Tadahiko Nakagawa, Tomoyuki Kawaguchi, Fumika Nakamura, Koichi Okamoto, Hiroshi Miyamoto, Masahiro Sogabe, Koichi Tsuneyama, Tetsuji Takayama

**Affiliations:** 1 Department of Gastroenterology and Oncology, Tokushima University Graduate School of Biomedical Sciences, 3-18-15 Kuramoto-Cho, Tokushima, 770-8503 Japan; 2 Department of Pathology and Laboratory Medicine, Tokushima University, 3-18-15 Kuramoto-Cho, Tokushima, 770-8503 Japan

**Keywords:** Ulcerative colitis, Inflammation

## Abstract

Although several angiogenesis-related factors are reportedly involved in the pathogenesis of ulcerative colitis (UC), the mechanisms by which they contribute to disease are unclear. We first examined the expression of angiogenesis-related factors in inflamed colorectal tissue of UC patients using antibody array, and identified the 5 factors with highest expression, which included matrix metalloproteinase-8, urokinase-type plasminogen activator (uPA), angiostatin/plasminogen, hepatocyte growth factor and endoglin. Subsequent real-time PCR experiments using additional colorectal tissues revealed that uPA mRNA levels were significantly higher in inflamed tissues than in non-inflamed tissues, and significantly correlated with the severity of UC. Mirror section immunohistochemistry revealed that uPA was expressed in the neutrophils of inflamed colorectal tissues. We administered dextran sulfate sodium (DSS) in drinking water to uPA knockout (uPA^−/−^) mice, and found that the disease activity index in uPA^-/-^ mice was marginally lower and the histological score in uPA^−/−^ mice was significantly lower than those in wild-type mice, suggesting the importance of uPA in colitis. When an uPA-selective inhibitor, UK122, was administered to DSS-treated C57BL6J mice, the disease activity index and histological score in those mice were significantly lower compared with control mice. Multiple cytokine/chemokine assay using colorectal tissues from uPA^−/−^ and UK122-treated mice revealed significantly lowered level of RANTES. In conclusion, uPA was highly expressed in neutrophils of the inflamed mucosa of UC patients, and the expression level correlated with the severity of UC. Genetic uPA deletion or pharmacological uPA blockade significantly ameliorated colitis in mice, concomitant with downregulation of RANTES.

## Introduction

Ulcerative colitis (UC) is a chronic idiopathic inflammatory bowel disease, the etiology of which is still poorly understood^1,2^. Inflammatory bowel disease (IBD) is traditionally regarded as a disease of westernized nations. However, the prevalence of UC is increasing worldwide, particularly in newly industrialized countries including Asian countries^3^. In the past decades, newly developed biologics and small molecules have been used to treat UC. However, many cases remain intractable, creating an unmet need to elucidate the etiology of UC and develop new treatments.

Angiogenesis is the process of new capillary formation from preexisting vasculature in adult tissue^4,5^. It is essential in physiological processes including embryogenesis, tissue growth, wound healing and the female reproductive cycle. Angiogenesis is also important in the pathology of inflammation because it can promote the migration of inflammatory cells to the site of inflammation, leading to the perpetuation of chronic inflammation^6^. Several pro-angiogenic factors are upregulated in the active colitis tissues of UC patients. Moreover, the proliferation, migration and adhesion of endothelial cells are dysregulated in colitis tissues, suggesting that abnormal angiogenesis in the colorectum is closely associated with the etiology or exacerbation of UC^7^. These findings suggest that inhibition of angiogenesis may be an effective treatment strategy for UC. In fact, some molecular targeted drugs approved for treatment of UC are related to angiogenesis. For example, TNF-α and IL-23 directly or indirectly promote angiogenesis^8,9^.

Therefore, in the present study, we first performed angiogenesis antibody array analysis using colonic tissue from UC patients. Because we ultimately found that urokinase-type plasminogen activator (uPA) is highly expressed in inflamed colonic tissue and that its expression levels correlated with the endoscopic severity score, we next investigated uPA expression and localization in colonic mucosa of UC patients by immunohistochemistry. We then determined whether uPA genetic deletion ameliorated intestinal inflammation or conferred protection against DSS-induced colitis in a murine model. Finally, we evaluated the efficacy of a uPA inhibitor in DSS-induced murine colitis model.

## Results

### Angiogenesis-related factors are increased in inflamed colorectal tissues of UC patients

**Figure 1:**
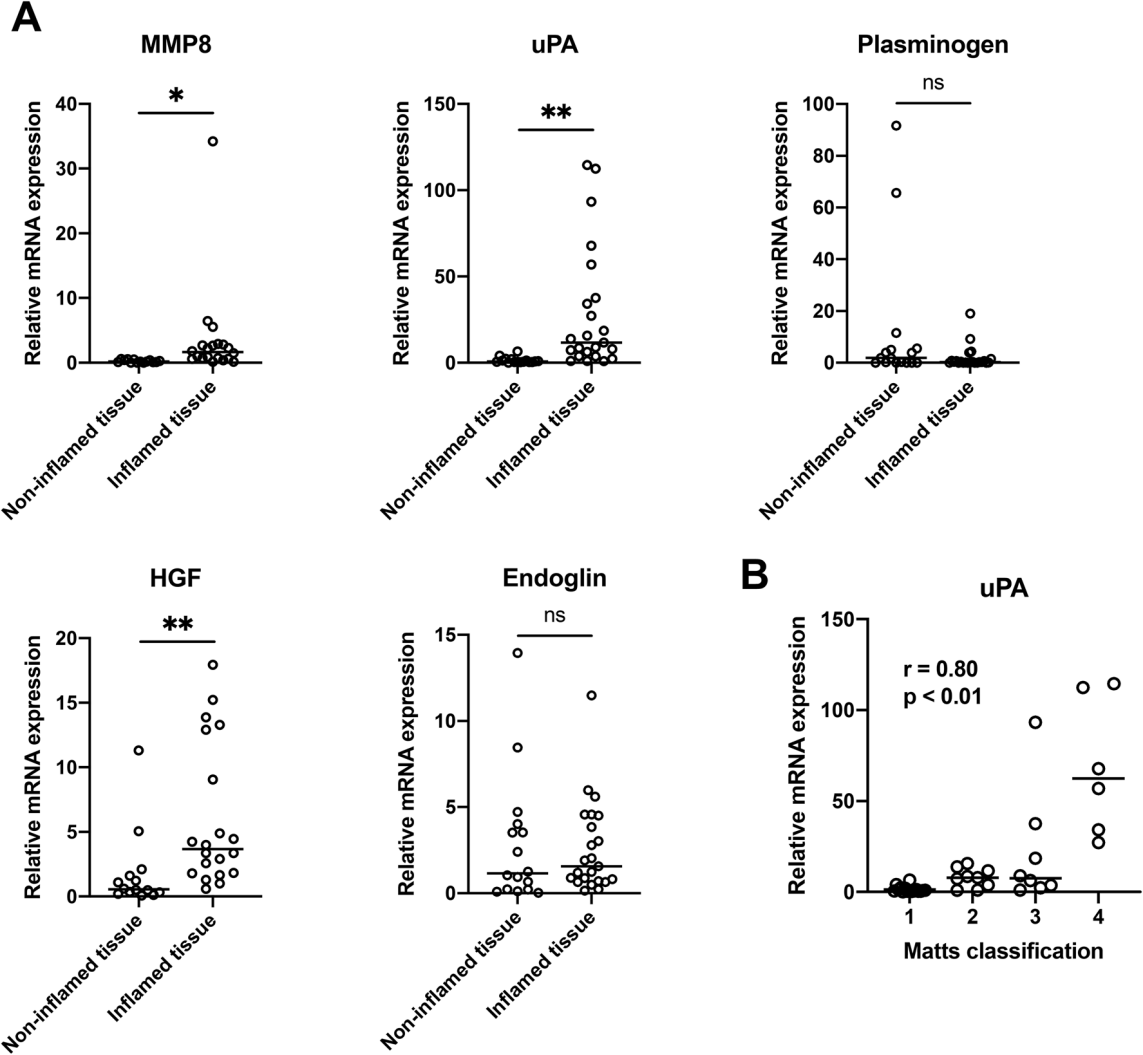
Expression of angiogenesis-related factors in inflamed or non-inflamed colorectal tissue of UC patients. (**A**) Levels of mRNA of five angiogenesis-related factors (MMP8, uPA, PLG, HGF, ENG) in the inflamed (n = 21) or non-inflamed (n = 15) colorectal tissue of UC patients were quantified by real-time PCR. Each bar represents the median value. *p < 0.05, **p < 0.01 by MannWhitney *U* test. (**B**) Endoscopic severity of disease in the colorectum assessed using Matts classification. Correlations between uPA levels and Matts classification in 36 specimens (21 inflamed tissue and 15 non-inflamed tissue) were analyzed using Spearman’s rank correlation test, *r* = 0.80, *p* < 0.01.

In order to investigate angiogenesis-related factors involved in UC, we first performed angiogenesis antibody array analyses using inflamed colorectal tissues from 6 UC patients and normal colorectal tissues from 3 healthy volunteers. We ranked factors based on their relative expression in inflamed versus normal tissue (Supplementary Figure. S1). The top five factors identified by this approach were MMP-8, uPA, angiostatin/plasminogen, HGF and endoglin. To validate the differential expression of each of these angiogenesis-related factors, we measured mRNA levels in 16 pairs of inflamed colorectal tissues and non-inflamed colorectal tissues and 5 inflamed colorectal tissues from UC patients. Because the latter 5 patients had severe inflammation throughout the colorectum, only inflamed tissue could be obtained. Of the five angiogenesis-related factors, levels of MMP-8, uPA and HGF mRNAs were significantly higher in inflamed colorectal tissues than in non-inflamed colorectal tissues (*p* < 0.05, *p* < 0.01 and *p* < 0.01 respectively; Fig. 1A). However, plasminogen and endoglin did not show statistically significant difference in mRNA levels between them. Furthermore, levels of uPA mRNA significantly correlated with the endoscopic severity score (*r* = 0.80, *p* < 0.01; Fig. 1B), whereas HGF mRNA levels correlated with the score very weakly with a low correlation coefficient. Therefore, we focused on uPA in the following experiments.

### uPA is expressed in neutrophils infiltrating into the colitis tissues of UC patients

**Figure 2:**
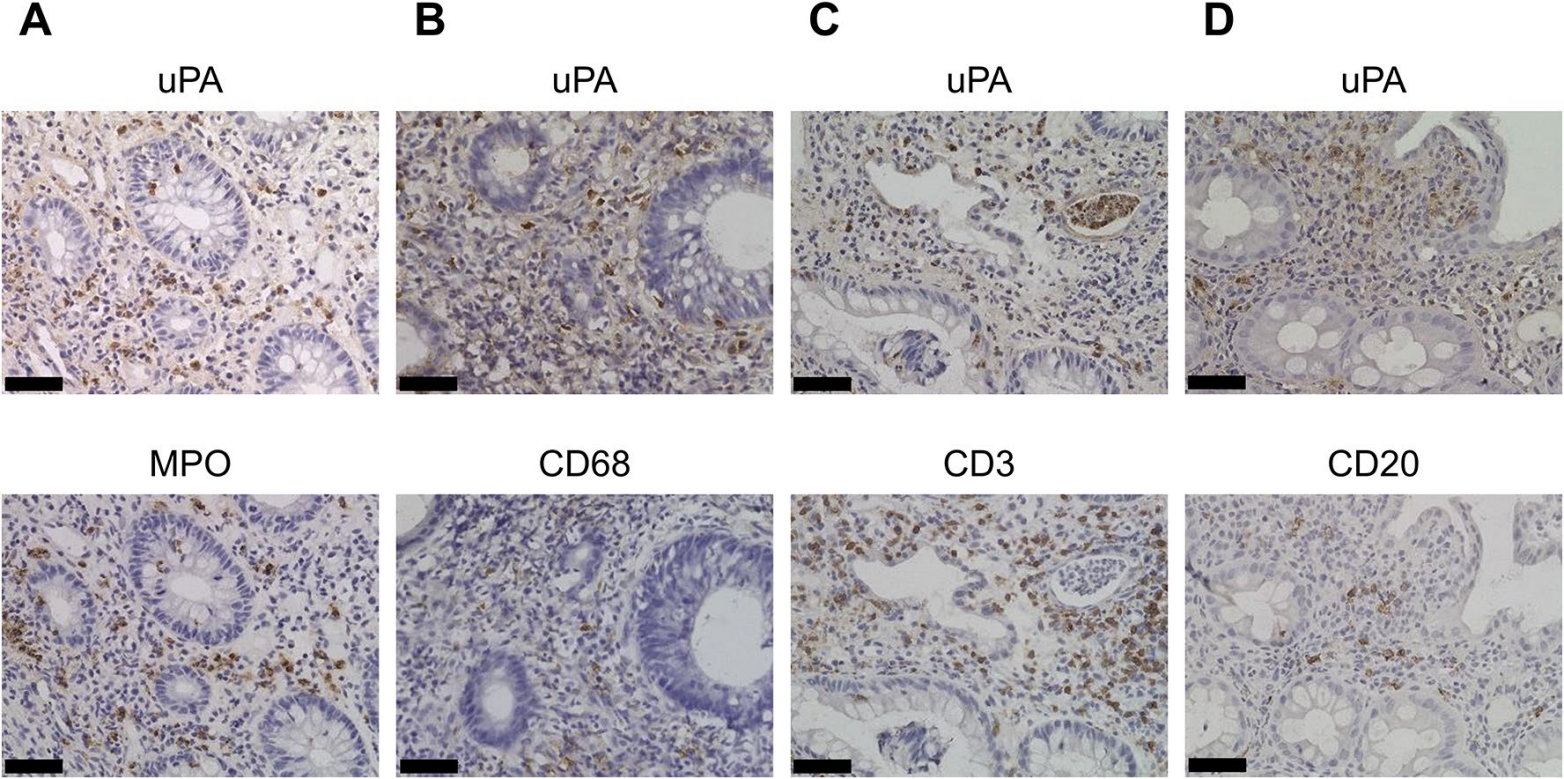
Representative immunohistochemical staining for uPA and MPO, CD68, CD3, or CD20 in inflamed colorectal tissue from UC patients using mirror sections obtained from two serial sections mounted on glass slides with the same cut surface facing upward. One section was stained using anti-uPA antibody and the other with anti-MPO (**A**), anti-CD68 (**B**), anti-CD3 (**C**), or anti-CD20 antibody (**D**). Each section was incubated with primary antibody and subsequently with polymer reagent and visualized by DAB chromogen. Scale bar, 50 μm.

To determine the localization of uPA expression in inflamed tissues of UC, we carried out immunohistochemical staining for uPA and MPO, CD68, CD3 or CD20 using mirror sections of colorectal tissues from UC patients. Strong cytoplasmic staining for uPA was observed in inflammatory cells, but not in epithelial cells. In the corresponding mirror section stained for neutrophil marker MPO, a nearly identical staining pattern to that for uPA was observed (Fig. 2A). However, the staining patterns of macrophage marker CD68, T cell marker CD3, and B cell marker CD20 respectively, in mirror sections were different from that of uPA (Fig. 2B,C,D). Moreover, double immunofluorescence revealed that uPA-positive cells were merged with MPO-positive cells, but not with CD68-, CD3- or CD20-positive cells (Supplementary Figure. S2). In addition, although only a few neutrophils were observed in colorectal tissues from normal subjects, the uPA-positive cells were merged with MPO-positive cells. These results indicate that uPA is expressed in neutrophils infiltrating into inflamed colorectal tissues.

### uPA^−/−^ mice exhibit milder DSS-induced colitis

**Figure 3:**
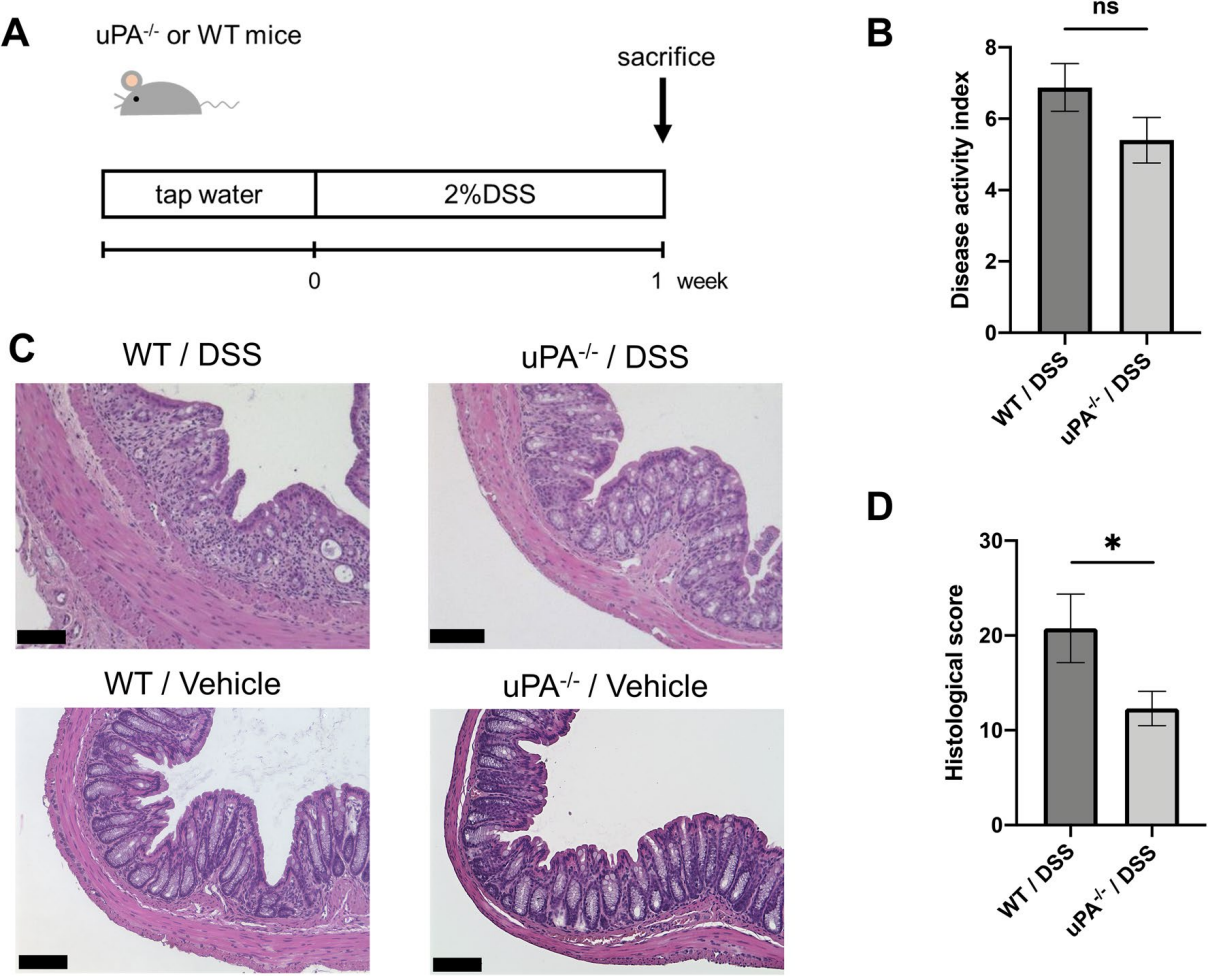
Effect of uPA knockout on DSS-induced colitis. (**A**) Male uPA^−/−^ mice (n = 10) and WT mice (n = 8) were administered 2% DSS in their drinking water for 1 week and sacrificed at day 8. (**B**) DAI was evaluated at day 8 by the scoring method of Cooper et al.^10^ (**C**) Representative H&E staining of colon sections from WT and uPA^-/-^ mice treated with DSS and control mice. Scale bar, 100 μm. (**D**) Histologic score of H&E staining image was evaluated by the scoring method of Williams et al.^11^ Data represent mean ± SEM. *p < 0.05 by Student’s *t* test.

To investigate the involvement of uPA in the pathogenesis of DSS-induced colitis, we administered 2% DSS to 10 uPA^**−**/**−**^ mice or 8 wild-type mice for 1 week, and compared symptomatic and histological findings between the 2 groups (Fig. 3A). The symptomatic disease activity index in uPA^**−**/**−**^ mice was marginally lower than in wild-type mice (5.20 ± 0.65 vs. 7.12 ± 0.77; *p* = 0.0710; Fig. 3B). Representative H&E staining images are shown in Fig. 3C. In the colorectal mucosa of wild-type mice treated with DSS, many inflammatory cells infiltrated into the mucosal layer and each crypt was markedly damaged in contrast with normal mucosa from wild-type mice treated with vehicle alone. However, far fewer inflammatory cells were observed in the mucosal layer and the crypts were morphologically preserved in uPA^**−**/**−**^ mice treated with DSS. The mean histological score based on H&E staining in uPA^**−**/**−**^ mice treated with DSS was significantly lower than in wild-type mice treated with DSS (12.30 ± 1.81 vs. 20.75 ± 3.61; *p* = 0.0406; Fig. 3D).

### uPA is expressed in neutrophils infiltrating into DSS-induced colitis tissue in mice

**Figure 4:**
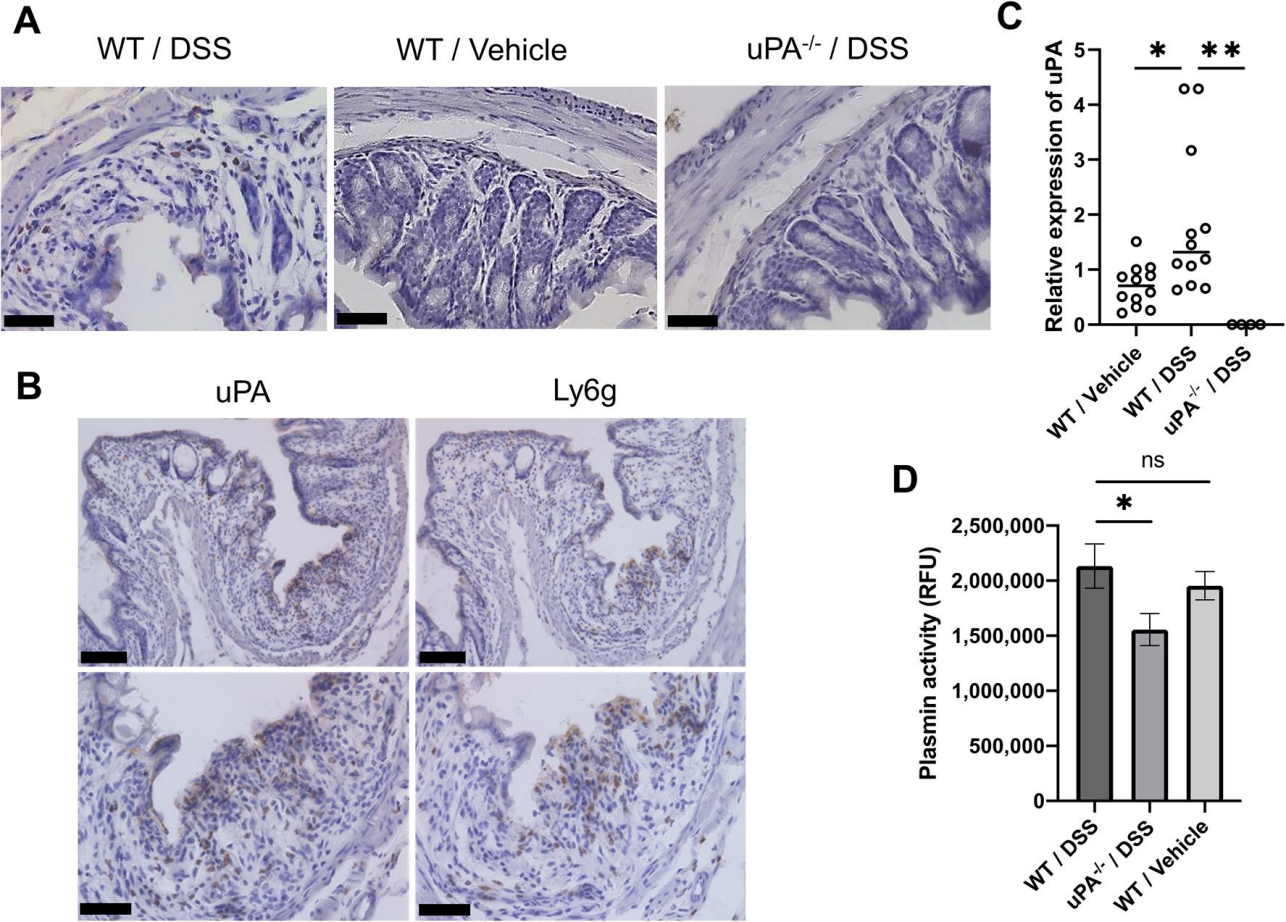
uPA expression and plasma plasmin activity of DSS-treated WT and uPA^−/−^ mice. (**A**) Immunohistochemistry for uPA was performed using colorectal tissues from DSS-treated or vehicle-treated WT mice and DSS-treated uPA^−/−^ mice (n = 3 per treatment group). The sections of colitis tissue were incubated with anti-uPA antibody, subsequently with polymer reagent and visualized using DAB chromogen. Scale bar, 50 μm. (**B**) Immunohistochemistry for uPA and Ly6g was performed using mirror sections from DSS-treated WT mice as described in the Materials and Methods. Scale bar, 100 μm (upper panel), 50 μm (lower panel). (**C**) Levels of uPA mRNA in colorectal tissue of DSS-treated (n = 12) or vehicle-treated (n = 12) WT mice and DSS-treated uPA^−/−^ mice (n = 4) were measured by real-time PCR. Each bar represents the median value. *p < 0.05, **p < 0.01 by Dunn’s multiple comparison test. (D) Plasmin activity in DSS-treated WT (n = 9) or uPA^−/−^ (n = 6) mice and vehicle-treated WT mice (n = 6) was measured using SensoLyte^Ⓡ^ AFC Plasmin Activity Assay Kit Fluorimetric. Data represent mean ± SEM. *p < 0.05 by Student’s *t* test.

To confirm uPA expression in DSS-induced colitis tissue of mice, we performed immunohistochemical staining for uPA in colorectal tissue from wild-type mice treated with DSS or vehicle, and from uPA^-/-^ mice treated with DSS. Representative staining patterns in colorectal tissues are shown in Fig. 4A. No uPA staining was observed in DSS-induced colitis tissues of uPA^**−**/**−**^ mice and in the normal-appearing colonic tissue of wild-type mice treated with vehicle alone, whereas uPA was clearly detected in DSS-induced colitis tissue of wild-type mice. The immunohistochemistry patterns for uPA and Ly6g in the mirror section of DSS-induced colitis tissue of wild-type mice were similar to one another (Fig. 4B). Similarly, double immune fluorescence revealed co-expression of uPA and Ly6g in infiltrating cells (Supplementary Figure. S3). These results suggest that uPA is expressed in neutrophils in DSS-induced colitis tissues of mice, similar to what we observed in inflamed tissues of UC patients. Moreover, the level of uPA mRNA in DSS-induced colitis tissue was significantly higher than in non-inflamed colorectal tissue of wild-type mice (1.32 vs. 0.71; *p* = 0.0388; Fig. 4C). We also confirmed that uPA was not detectable in DSS-induced colitis tissue of uPA^**−**/**−**^ mice.

Because uPA converts inactive plasminogen to plasmin, we measured plasmin activity in the plasma of DSS-treated wild-type and uPA^**−**/**−**^ mice. In the DSS-treated uPA^**−**/**−**^ mice, plasmin activity was significantly lower than in the DSS-treated wild-type mice (1.56 × 10^5^ ± 0.16 × 10^5^ vs. 2.13 × 10^5^ ± 0.20 × 10^5^; *p* = 0.0329; Fig. 4D). The plasma plasmin activity in DSS-treated wild-type mice was higher than in wild-type mice treated with vehicle alone, but the difference did not reach statistical significance (Fig. 4D). These data suggest that plasmin activity may be involved in the etiology or exacerbation of DSS-induced colitis although it does not necessarily serve as a sensitive biomarker for colorectal inflammation.

### uPA inhibitor ameliorates DSS-induced colitis in mice

**Figure 5:**
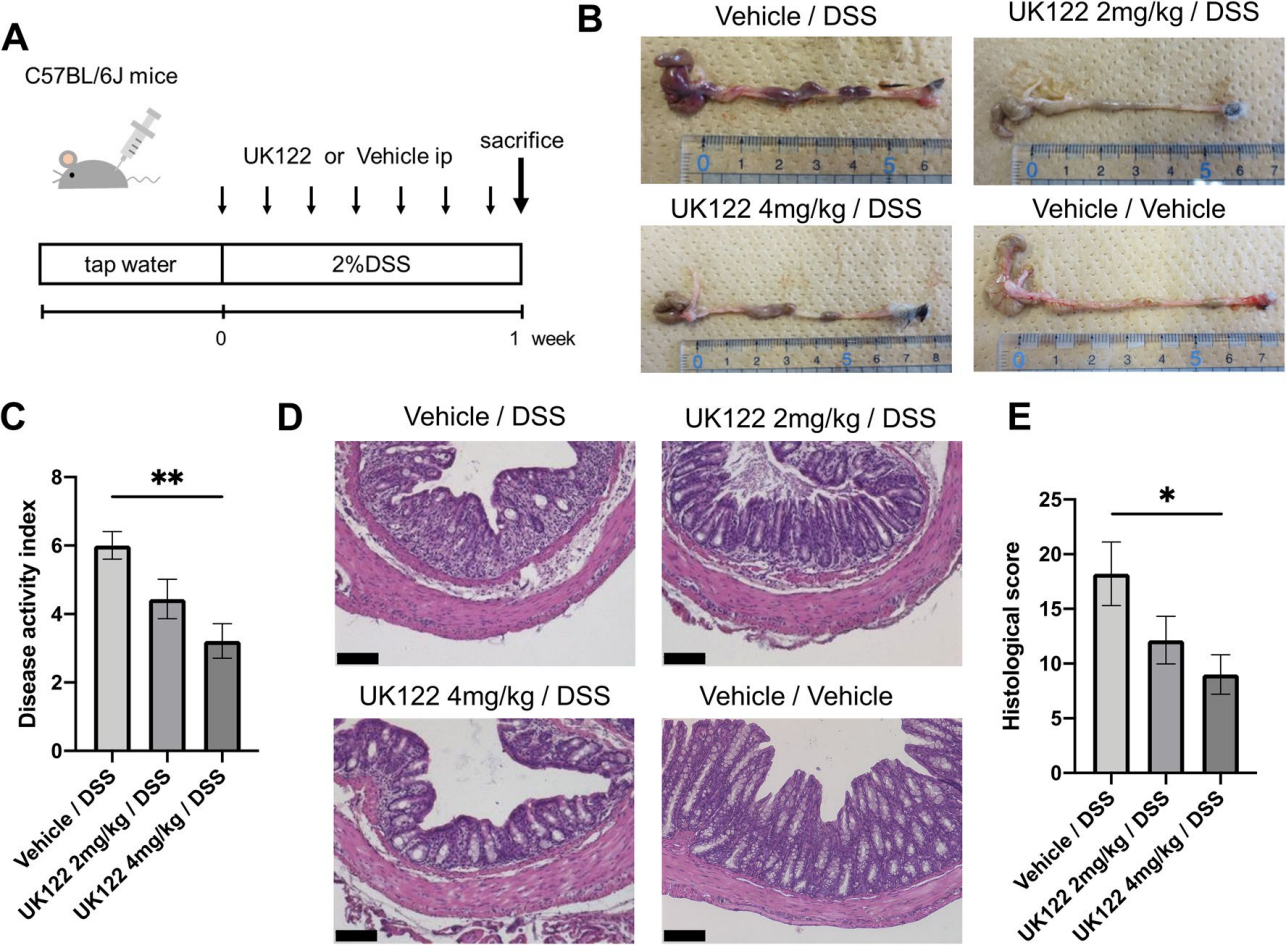
Effect of uPA inhibitor on DSS-induced colitis in C57BL/6 J mice. (**A**) Male mice were administered 2% DSS in their drinking water for 1 week, and intraperitoneally injected with 2 mg/kg or 4 mg/kg UK122 or vehicle from day 1 to day 7 (n = 18 per group). They were sacrificed at day 8. (**B**) Representative macroscopic images of the colon in each group. The lower right panel represents a colorectum of mice that did not receive DSS. (**C**) DAI was evaluated at day 8 by the scoring method of Cooper et al.^10^ (**D**) Representative H&E staining images of colon sections in each group. The lower right panel represents a colorectum of mice that did not receive DSS. Scale bar, 100 μm. (**E**) Histologic score of H&E staining image was evaluated by the scoring method of Williams et al.^11^ Data represent mean ± SEM. *p < 0.05, **p < 0.01 by Dunnett’s multiple comparison test.

To evaluate the therapeutic effect of uPA inhibitor UK122 on DSS-induced colitis, we intraperitoneally administered 2 mg/kg or 4 mg/kg of UK122 or vehicle alone to C57BL/6 J mice treated with DSS from day 1 to 7 (Fig. 5A). Representative macroscopic findings are shown in Fig. 5B. A considerable amount of bloody stool was observed in the colorectum of DSS-treated mice administered with vehicle alone. However, much less bloody stool was observed in DSS-treated mice administered with UK122 (both dose groups). The colorectum from DSS-treated mice administered with vehicle alone exhibited macroscopic findings of severe inflammation, whereas only mild inflammation was present in the colorectum of DSS-treated mice administered with UK122. The disease activity index was decreased by UK122 in a dose-dependent manner. There was a statistically significant difference between control mice and UK122 (4 mg/kg)-treated mice (6.00 ± 0.40 vs. 3.21 ± 0.50; *p* = 0.0008; Fig. 5C). The severity of colitis was further evaluated by histopathological analysis. Representative H&E staining images are shown in Fig. 5D. Strong inflammatory cell infiltration and crypt damage were observed in colorectal tissues of DSS-treated mice administered with vehicle alone. However, obviously less inflammatory cell infiltration was observed and the crypts were morphologically preserved in the colorectal tissues of DSS-treated mice administered with either dose of UK122. The histological score decreased in a dose-dependent manner following treatment with UK122 at 2 mg/kg and 4 mg/kg. There was a significant difference between control mice and UK122 (4 mg/kg)-treated mice (18.21 ± 2.91 vs. 9.00 ± 1.81; *p* = 0.0157; Fig. 5E).

### uPA receptor expression in inflamed colorectal tissues

Because uPA was overexpressed in inflamed colorectal tissues, we examined expression of uPA receptor (uPAR) in human and murine colorectum. The mRNA levels of uPAR in human inflamed colorectal tissues was significantly higher than in non-inflamed tissues (1.92 vs. 1.00; *p* = 0.0156; Supplementary Figure. S4). Similarly, uPAR mRNA levels in wild-type mice and uPA^−/−^ mice treated with DSS were significantly higher than in control mice (9.97 vs. 1.00; *p* = 0.0021, 7.80 vs. 1.48; *p* = 0.0224; Supplementary Figure. S4). The uPAR levels in uPA^−/−^ mice treated with DSS were lower compared with wild-type mice treated with DSS, but the difference did not reach statistical significance.

### Genetic and pharmacological inhibition of uPA reduces RANTES in colorectal tissues

**Figure 6:**
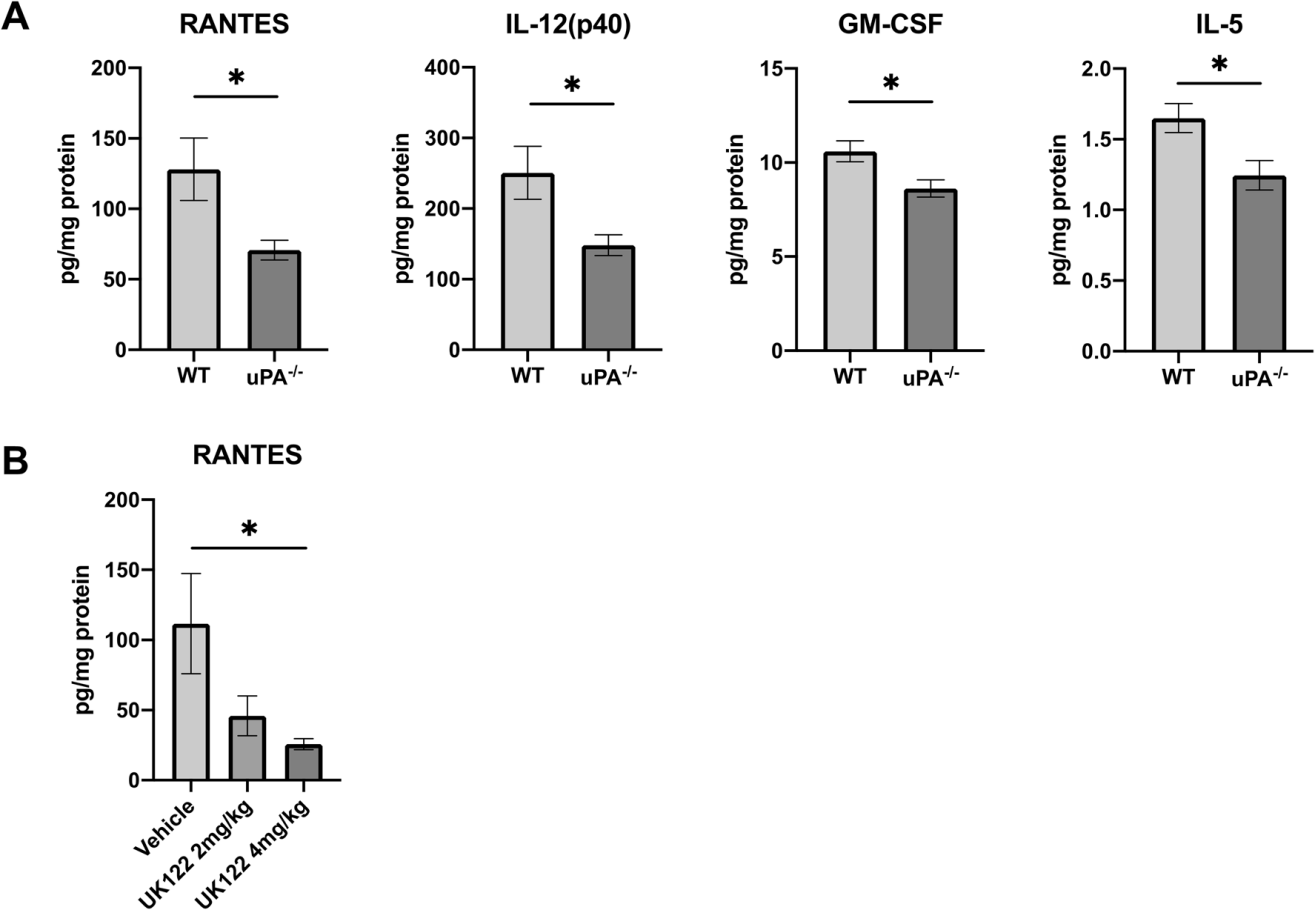
Cytokine expression in colorectal tissue from DSS-treated uPA^−/−^ mice, and C57BL/6 J mice intraperitoneally injected with UK122. (**A**) Cytokine concentration in the colorectal extracts of WT (n = 8) and uPA^−/−^ (n = 10) mice measured using Bio-Plex Pro Mouse Cytokine 23-Plex Assay kit in triplicate. (**B**) Cytokine concentration in the colorectal extracts of 8 mice treated with 2 mg/kg or 4 mg/kg UK122 or vehicle measured using Bio-Plex Pro Mouse Cytokine 23-Plex Assay kit in duplicate. Data represent mean ± SEM. *p < 0.05, by Student’s *t* test (**A**) and Dunnett’s multiple comparison test (**B**).

To assess the mechanism by which uPA deletion or inhibition protects against colitis, we measured the concentration of 23 cytokines in tissues from DSS-administered uPA^−/−^ mice and DSS-administered mice treated with UK122, and compared the results to the respective control mice. The concentrations of RANTES, IL-12(p40), GM-CSF and IL-5 in colorectal tissues of uPA^−/−^ mice were significantly lower than those in control mice (70.77 ± 7.02 vs. 128.04 ± 22.17; *p* = 0.0205 for RANTES, 148.26 ± 14.63 vs. 250.63 ± 37.47; *p* = 0.0178 for IL-12(p40), 8.63 ± 0.45 vs. 10.59 ± 0.56; *p* = 0.0147 for GM-CSF, 1.24 ± 0.10 vs. 1.64 ± 0.10; *p* = 0.0143 for IL-5; Fig. 6A). On the other hand, the concentration of RANTES decreased in a dose-dependent manner following treatment with 2 mg/kg or 4 mg/kg UK122. Only RANTES was significantly lower in mice treated with the higher dose of UK122 when compared to control mice (25.49 ± 3.86 vs. 111.64 ± 35.80; *p* = 0.0143; Fig. 6B). These data suggest that uPA deletion or inhibition protects against DSS-induced colitis by inhibiting RANTES production.

We then examined whether RANTES expression is induced by DSS treatment in wild-type mice and uPA^-/-^ mice. The RANTES mRNA level in wild-type mice treated with DSS was significantly higher than that in control mice treated with vehicle alone (4.74 vs. 1.00; *p* = 0.0341; Supplementary Figure. S5), consistent with the previous report^12^. On the other hand, the RANTES mRNA levels in uPA^−/−^ mice treated with DSS was higher than in uPA^−/−^ mice treated with vehicle alone, but no statistically significant difference was observed.

## Discussion

In this study, we found high uPA expression in the inflamed colorectal tissue of UC patients, and a positive correlation between the level of uPA and the severity of inflammation. We also demonstrated that the milder DSS-induced colitis in uPA^−/−^ mice was associated with downregulation of several inflammatory cytokines, suggesting an important role of uPA in UC. Moreover, a uPA-selective inhibitor significantly reduced the disease activity index and histological score of DSS-induced colitis in mice. This is the first study showing an anti-inflammatory effect of uPA deletion/inhibition on DSS-induced colitis in rodents, raising the possibility that uPA could be an ideal therapeutic target in UC.

uPA is a serine protease that converts plasminogen into plasmin. Moreover, uPA is involved in many physiological functions such as the immune response, chemotaxis, cell migration and activation of growth factors and cytokines. The binding of uPA with uPAR promotes plasminogen activation and signal transduction for various cell behaviors^13^. uPA is upregulated at the site of inflammation in chronic inflammatory diseases such as rheumatoid arthritis^14,15^, chronic obstructive pulmonary disease^16^ and psoriasis^17^. Moreover, uPA inhibition can reduce disease activity score in mouse arthritis models^18^. Although uPA was reportedly expressed in the inflamed mucosa of UC patients^19–21^, the cellular source of uPA in inflamed tissue and the relevance of uPA expression in colitis tissue have remained unclear. In the present study, we applied immunohistochemistry with mirror sections and double immunofluorescence to demonstrate that uPA was expressed mainly in MPO-positive neutrophils of human colitis tissue. Recently Almholt et al. reported that uPA is expressed in macrophages and neutrophils in the synovial membrane of patients with rheumatoid arthritis^18^. However, in this study, we showed that uPA is expressed in neutrophils but not in the macrophages that infiltrate into the colitis tissues of UC patients. Moreover, we found that levels of uPA expression correlated with endoscopic severity of UC. Additionally, we showed that uPA was expressed in Ly6g-positive neutrophils in a mouse model of DSS-induced colitis.

DSS-induced colitis was improved in uPA^−/−^ mice compared with control mice, indicating that uPA is an exacerbating factor for UC. Moreover, DSS-induced colitis was also ameliorated by treatment with a uPA-selective inhibitor, implying that uPA may be a therapeutic target in UC. Regarding the mechanism by which uPA inhibition reduces inflammation, we found that concentrations of RANTES, IL-12 (p40), GM-CSF and IL-5 in colitis tissue were significantly reduced in uPA^−/−^ mice. Of these 4 cytokines, the level of RANTES was also reduced in the DSS-induced colitis tissue in mice treated with the uPA inhibitor compared with control mice. RANTES is a so-called CC chemokine that contains 2 tandem cysteines in the N-terminus and has chemotactic activity for human CD4 memory T lymphocytes, monocytes, eosinophils and basophils. Since RANTES is upregulated in the colonic mucosa of UC patients^22,23^, it is plausible that the downregulation of RANTES upon uPA deletion/inhibition reduces the recruitment of leukocytes into the colitis tissue, and thereby ameliorates colitis in UC. In addition, uPA^-/-^ mice treated with DSS exhibit lower plasmin activity than wild-type mice treated with DSS. Recently, it was reported that plasmin inhibition attenuates experimental colitis in mice by suppressing matrix metalloproteinase 9-mediated cytokine release^24^. Moreover, uPA-activated plasmin stimulates NF-κB and production of cytokines, and is thus involved in exacerbation of inflammation^25–27^. Because NF-κB reportedly promotes mRNA transcription of RANTES^28,29^, genetic/pharmacologic blockade of uPA might decrease RANTES expression via inhibition of NF-κB. In addition, uPA-mediated activation of plasmin and matrix metalloproteinases plays a crucial role in extracellular matrix proteolysis, which is essential for the initiation of angiogenesis^30^. Considering these data, we suggest that inhibition of the plasminogen-plasmin system may contribute to amelioration of DSS-induced colitis in mice.

Eventually, we found that not only uPA but also uPAR was overexpressed in the inflamed colorectal tissues. The significance of uPAR expression in the pathogenesis of colitis remains controversial^31,32^, however our results suggest that increased uPA may contributes to exacerbation of inflammation of colorectal tissues together with increased uPAR. Moreover, recently, the soluble uPAR level in plasma has drawn attention as a biomarker for various diseases^33^. However, it is not necessarily a sensitive biomarker for colorectal inflammation, because no significant correlation was observed between plasma soluble uPAR levels and uPAR levels in colorectal tissue (Supplementary Figure.S4), consistent with previous report^34^.

A limitation of this study is that we have not directly shown the effect of RANTES on DSS-induced colitis. Although uPA has multiple functions including activation of the plasminogen-plasmin system, a causal relationship between uPA activity and RANTES expression has not yet been determined. Therefore, further study is necessary to examine the detailed role of RANTES and relationship between uPA and RANTES in UC.

In conclusion, we demonstrated that uPA is highly expressed in neutrophils of inflamed mucosa in UC patients and that uPA levels are positively correlated with the severity of UC. We also found that uPA gene deletion or pharmacological blockade of uPA activity ameliorated DSS-induced colitis in mice, concomitant with downregulation of RANTES expression. These data indicate that uPA plays an important role in inflammation of UC possibly through modulation of RANTES expression, and suggest that uPA may be a potential therapeutic target for the treatment of UC.

## Methods

### Patients

Six UC patients and 3 healthy volunteers were enrolled for the angiogenesis antibody array analysis. An additional 21 UC patients were enrolled for real-time PCR analysis of angiogenesis-related factors. Pairs of inflamed and non-inflamed colorectal tissues were obtained by endoscopic biopsy from 16 UC patients. Only inflamed tissue was obtained from 5 UC patients because they presented with severe inflammation representative of pancolitis, and almost no normal mucosa remained. In addition, 14 UC patients were enrolled for real-time PCR analysis of uPAR. Pairs of inflamed and non-inflamed colorectal tissues were obtained by endoscopic biopsy from 14 UC patients. All UC patients were attending Tokushima University Hospital. Baseline characteristics of the patients are described in Supplementary Table S1. Diagnoses were confirmed by clinical, endoscopic and histological criteria as defined by the Research Committee of IBD set up by the Japanese Ministry of Health, Labour and Welfare^35^. All UC patients were on medication including 5-aminosalicylic acid, corticosteroid, azathioprine, infliximab, vedolizumab, and ustekinumab. Endoscopic severity of the colorectum was assessed using the Matts classification^36^. This study was conducted in accordance with the Declaration of Helsinki and approved by the Ethics Committee of Tokushima University Hospital. Written informed consent was obtained from all patients prior to colonoscopy.

### Angiogenesis antibody array

The expression profile of angiogenesis-related proteins in colorectal tissue of UC patients and healthy volunteers was analyzed using the Proteome Profiler Human Angiogenesis Array Kit (R&D Systems, Minneapolis, MN). The colorectal tissues were homogenized in PBS with protease inhibitor and 1% Triton X 100. The total protein concentration was determined using a Pierce BCA Protein Assay Kit (Thermo Fisher Scientific, Waltham, MA). The procedure was performed according to the manufacturer’s protocol. The supernatant was incubated with membranes provided in the kit. The membranes were then exposed to X-ray films and the average signal of pairs of duplicate spots was determined.

### uPA knockout mice

B6.129S2-Plau^tm1Mlg^/J (uPA^−/−^) mice were purchased from the Jackson Laboratory (Bar Harbor, ME). Nine to 10 week-old, age matched, male uPA^−/−^ mice and their control littermates were used for the experiments. For the induction of colitis, mice were administered 2% dextran sulfate sodium (DSS, Wako Pure Chemical Industries, Osaka, Japan) in the drinking water for 7 days. Mice were anesthetized with isoflurane and then sacrificed by cervical dislocation at day 8. All experimental procedures in this study were approved by the Animal care and Use Committee of Tokushima University and performed in compliance with the guidelines of the Committee of Animal Care and Use of Tokushima University and ARRIVE guidelines.

### Mouse colitis model and uPA inhibition

Eight week-old C57BL/6 J male mice were purchased from CLEA Japan (Tokyo, Japan). For induction of colitis, mice were administered 2% DSS in drinking water for 7 days. The uPA-selective inhibitor UK122 (Santa Cruz Biotechnology, Dallas, TX) was dissolved in dimethyl sulfoxide (DMSO) and saline. Mice were intraperitoneally injected with 2 mg/kg or 4 mg/kg of UK122 or vehicle once per day from day 1 to 7. Mice were anesthetized with isoflurane and then sacrificed by cervical dislocation at day 8.

### Assessment of colitis in mice

Severity of colitis was evaluated at day 7, using the disease activity index (DAI) previously described by Cooper et al^10^. The scoring system includes weight loss (0, none; 1, 1–5%; 2, 5–10%; 3, 10–15%; 4, 15–20%), stool consistency (0, normal; 2, loose stool; 4, diarrhea) and fecal blood (0, normal; 2, Hemoccult; 4, gross bleeding). Colorectal tissues were removed, fixed with 4% paraformaldehyde and embedded in paraffin. After cutting slices, thin tissue sections were stained with hematoxylin and eosin (H&E). Histology was evaluated using the scoring system previously described by Williams et al.^11^, which includes inflammation severity (0, none; 1, mild; 2, moderately; 3, severe), inflammation extent (0, none; 1, mucosa; 2, mucosa and submucosa; 3, transmural) and crypt damage (0, none; 1, basal 1/3 damaged; 2, basal 2/3 damaged; 3, crypts lost and surface epithelium present; 4, crypts and surface epithelium lost). Histologic score was calculated by multiplying the first three parameters by their percentage involvement, giving a maximum score of 40.

### TaqMan real-time PCR

Total RNA was extracted from biopsy specimens of UC patients and murine colorectal tissue using RNeasy Mini kit (Qiagen, Hilden, Germany). TaqMan real-time PCR was performed as previously described^37^. All gene expression was normalized to 18S ribosomal RNA. The following primer sets (Thermo Fisher Scientific, Waltham, MA) were used: human urokinase-type plasminogen activator (PLAU/uPA, Hs01547054_m1), matrix metalloproteinase-8 (MMP-8, Hs01029057_m1), plasminogen (PLG, Hs00264877_m1), hepatocyte growth factor (HGF, Hs00300159_m1), endoglin (ENG, Hs00923996_m1), 18S (Hs99999901_s1), urokinase-type plasminogen activator receptor (PLAUR, Hs00959822_m1), murine urokinase-type plasminogen activator (Plau/uPA, Mm00447054_m1), urokinase-type plasminogen activator receptor (Plaur, Mm00440913_m1), RANTES (Ccl5, Mm01302427_m1), and Rn18s (Mm03928990_g1).

### Immunohistochemistry

Immunohistochemical staining of human and murine colitis tissue was performed using the polymer method. Briefly, frozen sections were washed, and endogenous peroxidase was inactivated by incubation with 0.3% H_2_O_2_ in methanol for 1 h. Sections were blocked with blocking solution (Dako, Glostrup, Denmark) for 30 min at room temperature. For human colon sections, rabbit anti-uPA (17968–1-AP, Proteintech, Rosemont, IL), mouse anti-MPO (sc-365436, Santa Cruz), mouse anti-CD68 (ab955, Abcam, Cambridge, UK), mouse anti-CD3 (ab699, Abcam) and mouse anti-CD20 (ab9475, Abcam) were used as primary antibodies. For mouse colon sections, rabbit anti-uPA (17968–1-AP) and rat anti-Ly6g (ab253779, Abcam) were used as primary antibodies. Subsequently, the human colon sections were incubated with polymer reagent (anti-mouse IgG and anti-rabbit IgG, ChemMate Envision, K5027, Dako). Mouse colon sections were incubated with another polymer reagent (anti-rabbit IgG, MP-7401; anti-rat IgG, MP-7444, Vector Laboratories, Burlingame, CA). Finally, sections were visualized with diaminobenzidine (DAB) chromogen, and counterstained with Mayer’s hematoxylin. To investigate the localization of uPA in the inflamed tissue, mirror sections were obtained using two serial sections mounted on glass slides with the same cut surface facing upward; one section was stained using anti-uPA antibody and the other was stained for cell markers.

### Double immunofluorescence staining

Double immunofluorescence staining for human colitis tissue of UC patients, normal colon tissue of healthy volunteers, and murine colitis tissue was performed. Frozen sections were treated with citrate buffer (pH 6.0) for 10 min at 100 °C for antigen retrieval and then blocked with blocking solution for 30 min at room temperature. Sections were incubated with a mixture of 2 primary antibodies overnight at 4 °C. The sections were then incubated with a mixture of 2 secondary antibodies for 1 h at room temperature. The following secondary antibodies were applied: Alexa Fluor 488-conjugated goat anti-mouse IgG (H + L), F(ab’)_2_ Fragment (#4408, Cell Signaling Technology, Danvers, MA), Alexa Fluor 555-conjugated goat anti-rabbit IgG (H + L), F(ab’)_2_ Fragment (#4413, Cell Signaling Technology), and Alexa Fluor 488-confugated goat anti-rat IgG (H + L) (#4416, Cell Signaling Technology). Nuclei were counterstained with 4’,6-diamidino-2-phenylindole (DAPI). The stained sections were observed using a BZ-X710 fluorescence microscope (Keyence, Osaka, JPN).

### Measurement of colon cytokines

For cytokine measurement, mouse colonic tissue lysate was extracted using Bio-Plex Cell Lysis Kits (Bio-Rad, Hercules, CA) according to the manufacturer’s protocol. In brief, dissected colorectal tissues were homogenized in cell lysis buffer containing phosphatase and protease inhibitors. The homogenized solution was centrifuged for 15 min at 12,000 rpm, and the supernatant was separated into aliquots and frozen at − 80 °C until analysis. The protein concentration was determined using Pierce BCA Protein Assay Kits. Cytokine concentrations were measured using Bio-Plex Pro Mouse Cytokine 23-Plex Assay kits (Bio-Rad) according to the manufacturer’s protocol.

### Plasmin activity assay

Plasmin activity in mouse plasma was measured using a SensoLyte^Ⓡ^ AFC Plasmin Activity Assay Kit (AnaSpec, San Jose, CA). The assay was performed according to the manufacturer’s protocol. Briefly, plasma diluted 1:10 in PBS was added to 96-well plates and incubated with plasmin substrate for 1 h at 37 °C. Plasmin substrate releases the AFC (7-amido-4-trifluoromethylcoumarin) fluorophore upon plasmin cleavage. Fluorescence intensity was measured at excitation/emission = 380 nm/500 nm using a microplate reader (Spectra Max i3, Molecular Devices, San Jose, CA).

### Statistics

Levels of mRNA in inactive UC and active UC were compared using Mann–Whitney *U* tests. Correlations between levels of mRNA and the Matts classification were calculated using Spearman’s rank correlation test. Levels of mRNA in experiments involving knockout mice were compared using Dunn’s multiple comparison test. Disease activity index, histological score, plasmin activity and cytokine concentration in experiments involving knockout mice were compared using Student’s *t* test. Disease activity index, histological score and cytokine concentration in experiments on uPA inhibition were compared using Dunnett’s multiple comparison test. GraphPad Prism version 8.0 (GraphPad Software, San Diego, CA) was used for this analysis. A *p* value less than 0.05 was considered significant.

## Supplementary Information

Supplementary Information.

## Data Availability

The data underlying this article are available on reasonable request to the corresponding author.
