# Supplementary material for: Urokinase-type plasminogen activator blockade ameliorates experimental colitis in mice
Source: Sci Rep. 2023 Feb 18;13:2899. doi: 10.1038/s41598-023-29824-1 (PMC9938860; doi:10.1038/s41598-023-29824-1)
Supplement: Supplementary file 1 — Supplementary Information. [file 41598_2023_29824_MOESM1_ESM.pdf]

Supplementary Information

Urokinase-type plasminogen activator blockade ameliorates experimental colitis in mice

Yoshifumi Kida[1], Toshiya Okahisa[1], Yasushi Sato[1], Masahiro Bando[1], Shota Fujimoto[1], Beibei Ma[1], Tadahiko Nakagawa[1], Tomoyuki Kawaguchi[1], Fumika Nakamura[1], Koichi Okamoto[1], Hiroshi Miyamoto[1], Masahiro Sogabe[1], Koichi Tsuneyama[2], Tetsuji Takayama[1, *]

1) Department of Gastroenterology and Oncology, Tokushima University Graduate School of Biomedical Sciences, 3-18-15 Kuramoto-cho, Tokushima 770-8503, Japan

2) Department of Pathology and Laboratory Medicine, Tokushima University, 3-18-15 Kuramoto-cho, Tokushima, 770-8503, Japan

Address correspondence to: Tetsuji Takayama, MD, PhD, 3-18-15, Kuramoto-cho, Tokushima, 770-8503, Japan. Tel: +81-88-633-7124. Fax: +81-88-633-9235.

E-mail: takayama@tokushima-u.ac.jp

Supplementary Methods

Measurement of Plasma suPAR

Human plasma levels of suPAR were determined using a human suPAR ELISA Kit (E-EL-H2584, Elabscience, Houston, TX), according to the manufacturer's protocol. This ELISA kit utilizes the sandwich ELISA technique. Briefly, standards and samples were added to microplate wells pre-coated with captured antibody and incubated for 1 h at 37˚C. After washing, a biotinylated antibody was added to the wells and incubated for 1 h at 37˚C, followed by addition of an HRP-conjugated antibody and incubation for 30 min at 37˚C. A substrate was added to the wells and incubated for 15 min at 37˚C and then a stop solution was added to the wells. Optical density at 450 nm was measured using a microplate reader (Spectra Max i3, Molecular Devices, San Jose, CA). Mouse plasma levels of suPAR were determined using a mouse suPAR ELISA kit (MBS7256369, MyBioSource, San Diego, CA), according to the manufacturer's protocol. This ELISA kit utilizes the competitive ELISA technique. Briefly, standards and samples were incubated together with SuPAR-HRP conjugate in pre-coated microplate wells for 1 h. After washing, a substrate for HRP was added to the wells and incubated for 15 min at 37˚C. Finally, a stop solution was added and optical density at 450 nm was measured using a microplate reader (Spectra Max i3).

Supplementary Figure Legends

Supplementary Figure S1

Ratio of mucosal concentration of angiogenesis-related factors in the UC patients to healthy controls**.** Expression level of angiogenesis-related factors in colorectal tissue of UC patients (n = 6) and healthy controls (n = 3) was quantified by antibody array. Data represent mean ± SEM.

Supplementary Figure S2

Representative double immunofluorescence staining for uPA and MPO, CD68, CD3, or CD20 in inflamed colorectal tissue from UC patients and normal colorectal tissue from healthy controls. Frozen sections from UC patients were stained using anti-uPA antibody and anti-MPO (A), anti-CD68 (B), anti-CD3 (C), or anti CD20 antibody (D). Frozen sections from healthy controls were also stained using anti-uPA antibody and anti-MPO (E), anti-CD68 (F), anti-CD3 (G), or anti CD20 antibody (H). Each section was incubated with primary antibody and subsequently with fluorescence conjugated secondary antibody. Arrowheads denote co-labeled cells. Scale bar, 20 μm.

Supplementary Figure S3

Representative double immunofluorescence staining for uPA and Ly6g in mouse colitis tissue. Frozen sections from DSS-induced colitis mice were stained using anti-uPA antibody (A) and anti-Ly6g antibody (B) and merged (C). Arrowheads denote colabeled cells. Scale bar, 20 μm.

Supplementary Figure S4

uPAR expression in colorectal tissue and plasma suPAR concentration in UC patients and mice. (A) The mRNA levels of uPAR in inflamed or non-inflamed colorectal tissue of UC patients were quantified by real-time PCR ($n = 14$ per group). Each bar represents the median value. *$p < 0.05$ by Mann-Whitney U test. (B) The mRNA levels of uPAR in colorectal tissue of DSS-treated ($n = 11$) or vehicle-treated ($n = 7$) WT mice and DSS-treated ($n = 11$) or vehicle-treated ($n = 7$) uPA$^{-/-}$ mice were quantified by real-time PCR. Each bar represents the median value. *$p < 0.05$, **$p < 0.01$ by Dunn's multiple comparison test. (C) Plasma suPAR concentration in UC patients ($n = 26$) and healthy controls (HC) ($n = 15$) was measured using an ELISA. (D) Plasma suPAR concentration of DSS-treated or vehicle-treated WT mice and DSS-treated or vehicle-

treated uPA$^{-/-}$ mice was measured using an ELISA (n = 7 per group).

Supplementary Figure S5

RANTES expression in colorectal tissue of mice. The mRNA levels of RANTES in colorectal tissue of DSS-treated (n = 11) or vehicle-treated (n = 7) WT mice and DSS-treated (n = 11) or vehicle-treated (n = 7) uPA$^{-/-}$ mice were quantified by real-time PCR. Each bar represents the median value. *$p < 0.05$ by Dunn's multiple comparison test.

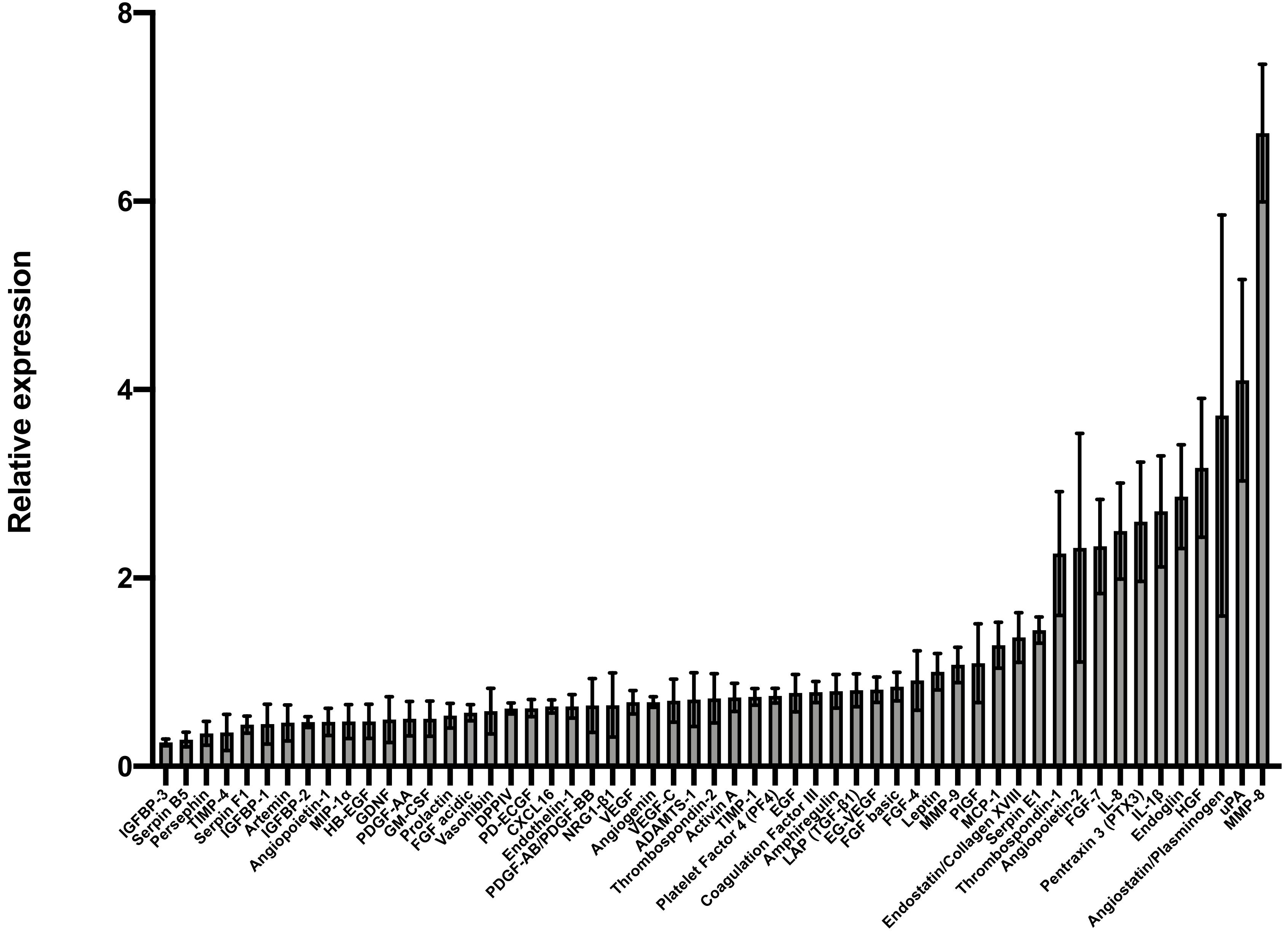

Supplementary Figure S1

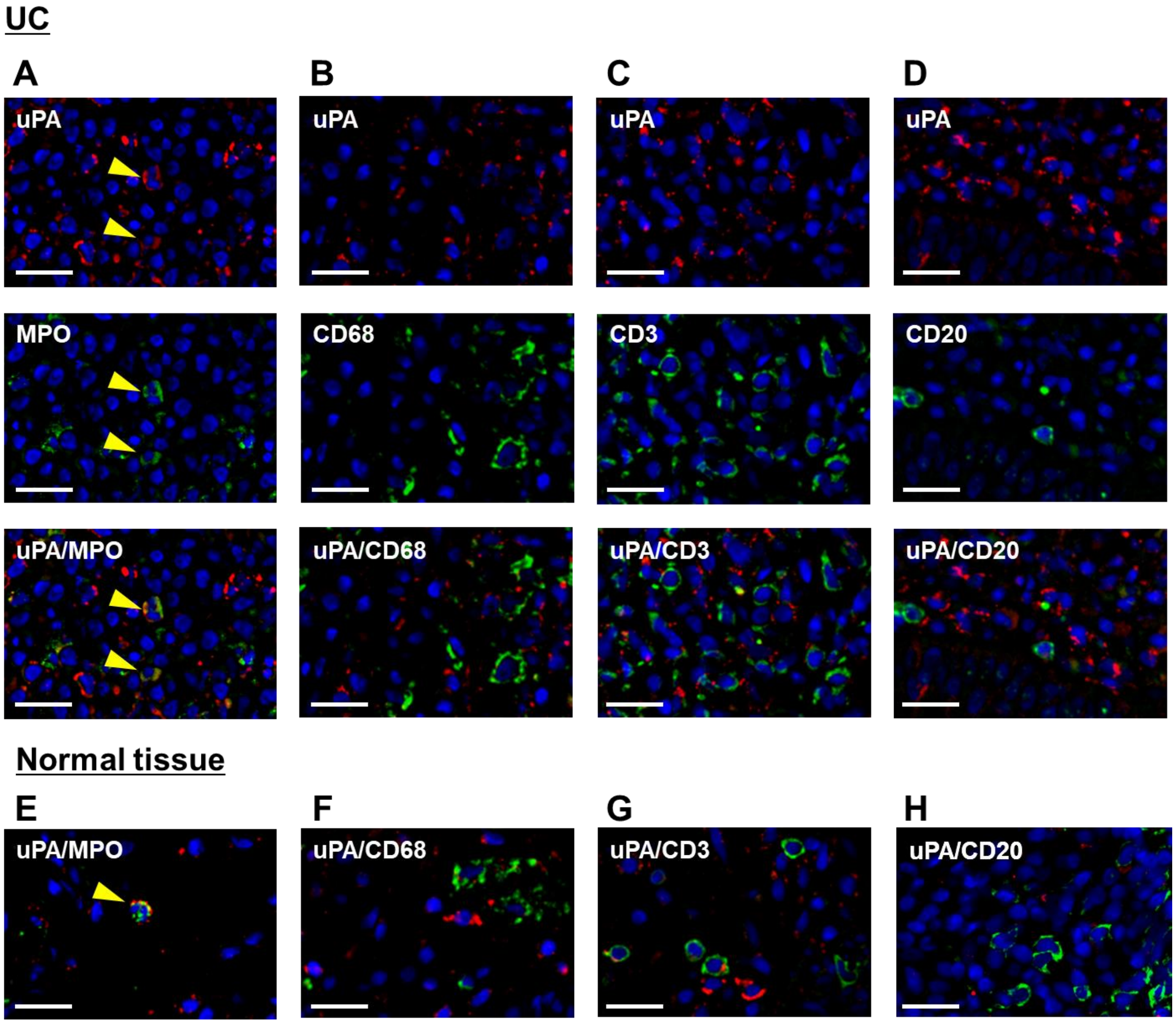

Supplementary Figure S2

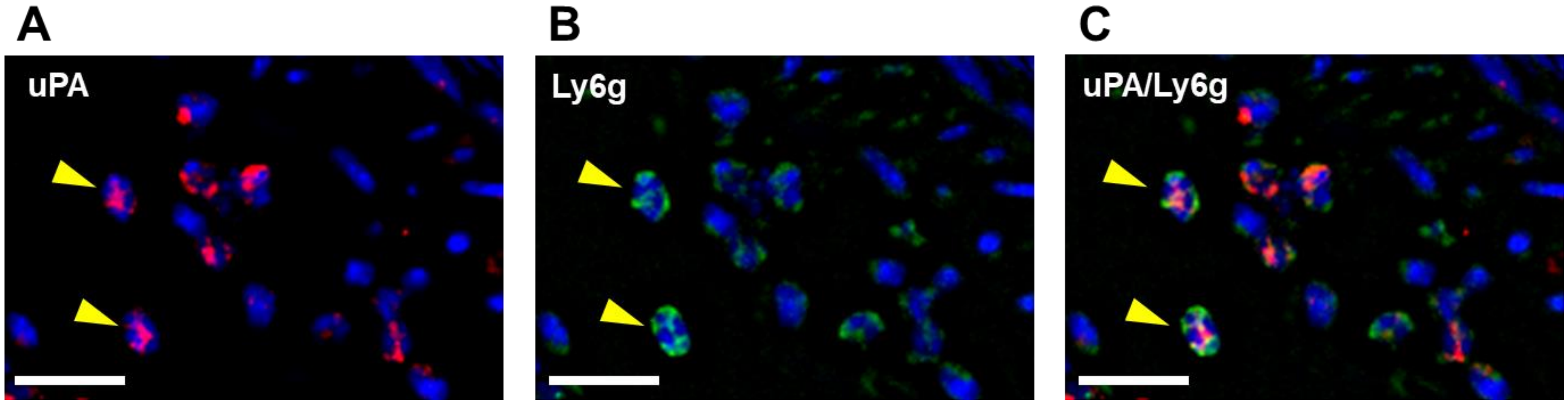

Supplementary Figure S3

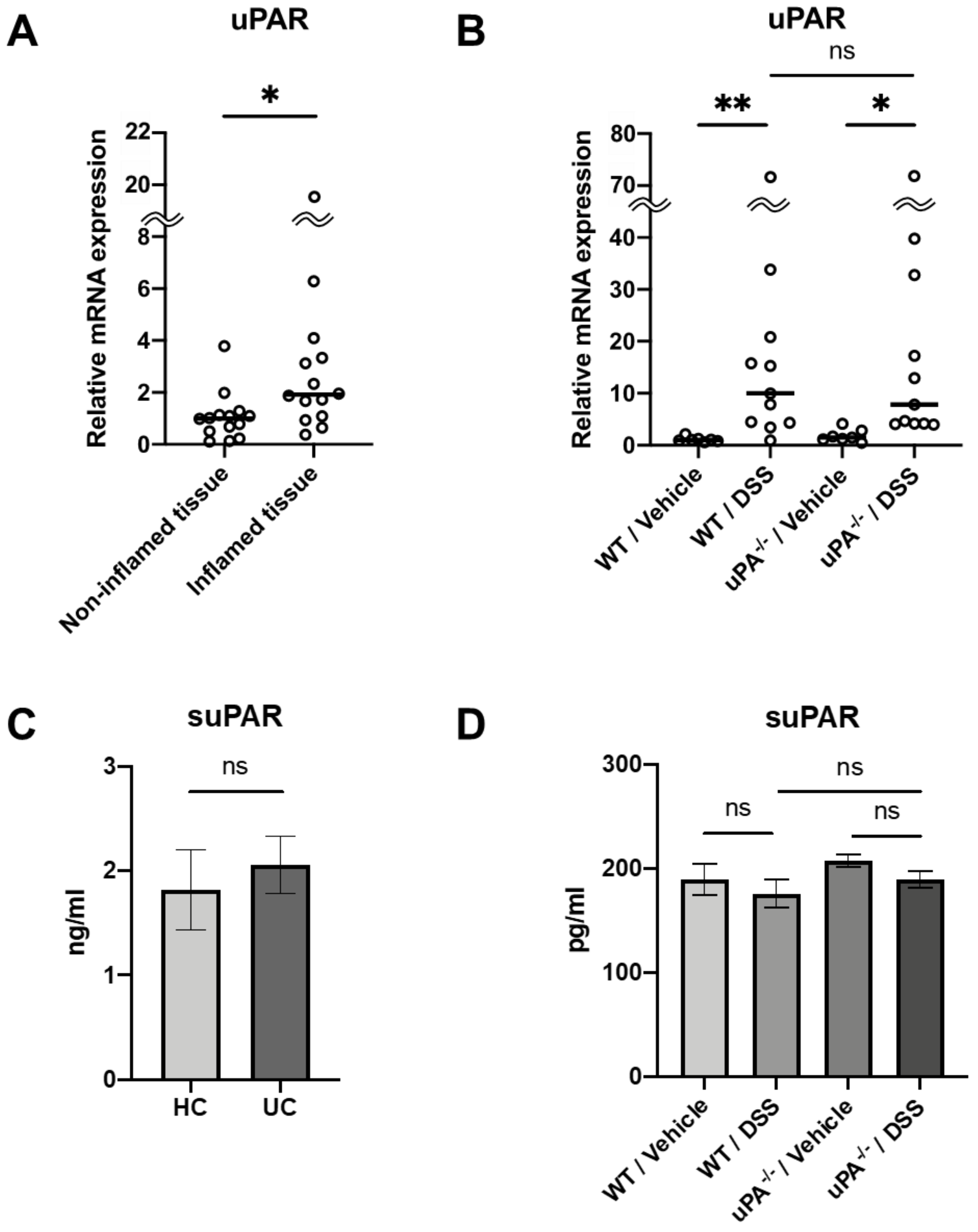

Supplementary Figure S4

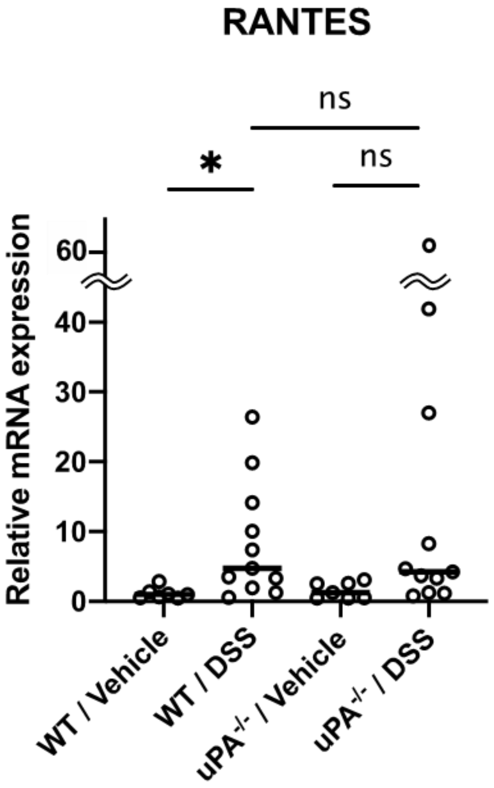

Supplementary Figure S5

Supplementary Table S1: Baseline characteristics of ulcerative colitis patients

Characteristics	UC Patients		
	Patients for antibody array analysis[a]	Patients for real-time PCR of angiogenesis-relatred factor[b]	Patients for real-time PCR of uPAR[b]
Number of patients	6	21	14
Male/Female	3/3	16/5	6/8
Age (years; median)	49	47	65
Duration of disease (years; median)	12.5	8	9
Mayo score (median)	4	4	6
Extent of UC			
Proctitis	2	4	0
Left-side colitis	1	6	2
Pancolitis	3	11	12
Medications			
5-aminosalicylic acid	4	19	14
Corticosteroid		3	3
Azathioprine	1	4	4
Infliximab		1	2
Vedolizumab			1
Ustekinumab			1

[a] One biopsy specimen was taken from active regions under colonoscopy.

[b] Two biopsy specimens were taken under colonoscopy; 1 for each from active and inactive regions.
